# Supplementary material for: IL-4 Signaling Promotes Myoblast Differentiation and Fusion by Enhancing the Expression of MyoD, Myogenin, and Myomerger
Source: Cells. 2023 Apr 29;12(9):1284. doi: 10.3390/cells12091284 (PMC10177410; doi:10.3390/cells12091284)
Supplement: Supplementary file 1 [file cells-12-01284-s001.zip › cells-2307640-supplementary.pdf]

IL-4 Signaling Promotes Myoblast Differentiation and Fusion by Enhancing the Expression of MyoD, Myogenin, and Myomerger

Mitsutoshi Kurosaka [1,*], Yung-Li Hung [2], Shuichi Machida [2,3] and Kazuhisa Kohda [1]

List of Supplementary Material

Figure S1. SiRNA-mediated inhibition of IL-4Rα expression

Figure S2. Myomerger transfection restored myomerger of expression in IL-4RαKD C2C12 cells

Figure S3. SiRNA-mediated inhibition of NFATc2 expression

Table S1. Primer sequences used in qRT-PCR

Figure S1. SiRNA-mediated inhibition of IL-4Rα expression

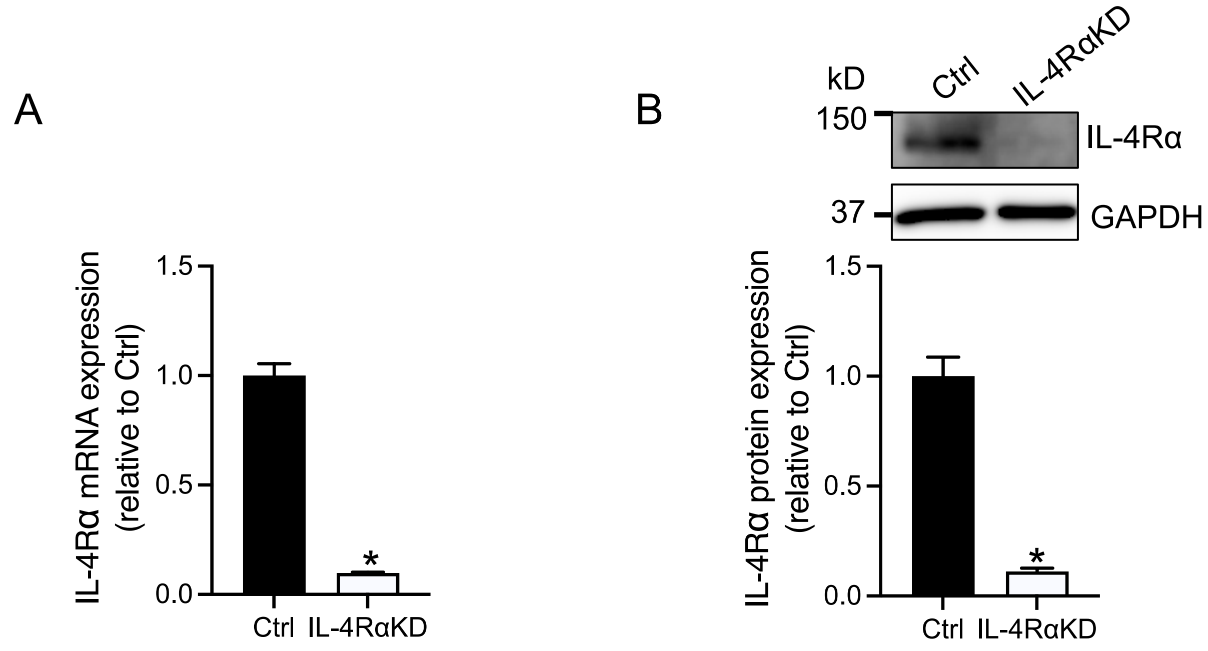

C2C12 myoblasts were transfected with control (Ctrl) or IL-4Rα siRNA (IL-4RαKD) and were maintained in GM for 24 h. Cells were then collected and processed for qRT-PCR or Western blot analysis. Fold changes of IL-4Rα mRNA and protein levels are shown in **(A)** and **(B)**, respectively. N = 3 per group. * $P < 0.05$. Data are presented as mean ± SD.

Figure S2. Myomerger transfection restored myomerger of expression in IL-4RαKD C2C12 cells

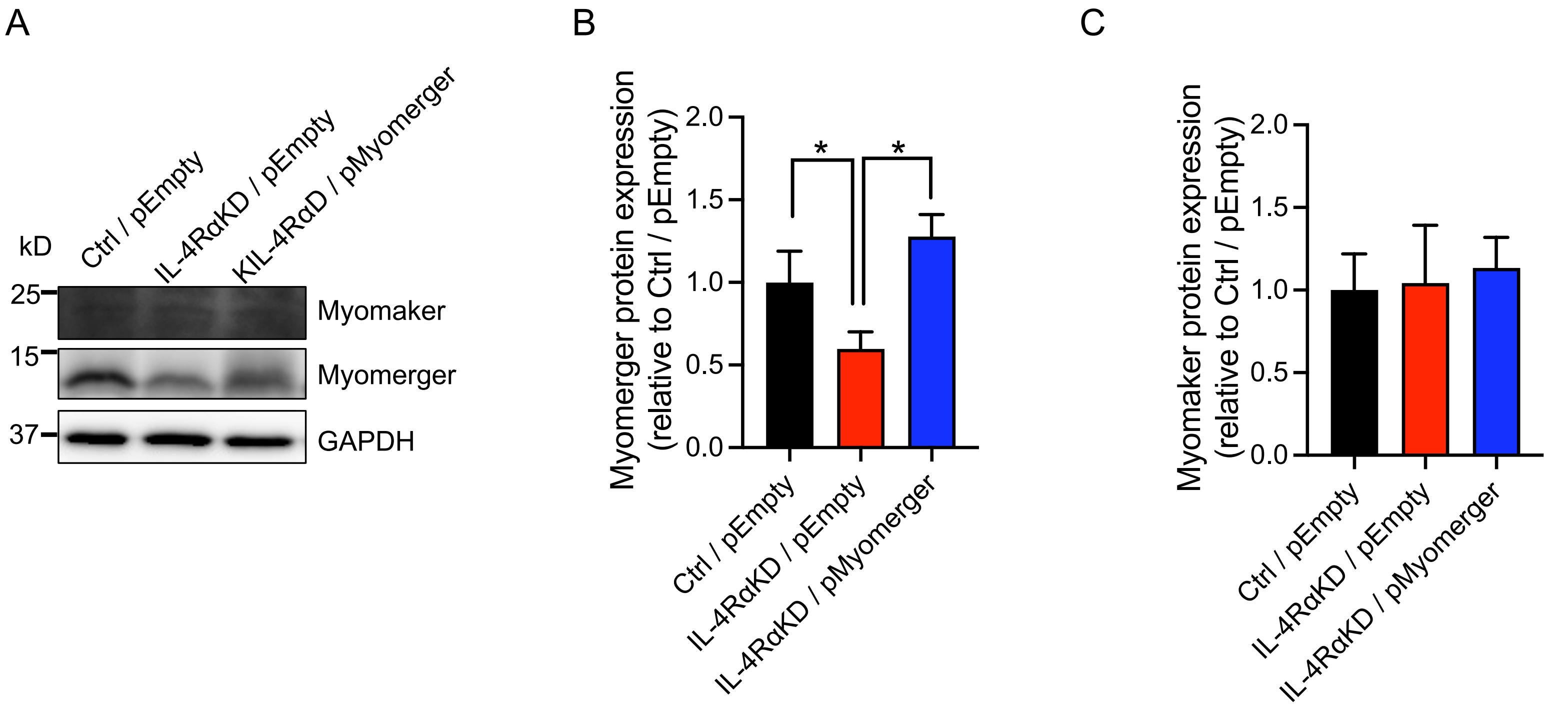

(A) pMyomerger, a myomerger expression vector, or pEmpty, a control vector, was transfected into control or IL-4RαKD C2C12 cells. Representative Western blot images are shown. **(B)** and **(C)** The diagrams show quantitative analysis of myomerger and myomaker protein expression. Myomerger transfection restored myomerger expression in the IL-4RαKD/pMyomerger group, while the expression level of myomaker was comparable among the three groups. N = 4 per group. Data are presented as mean ± SD. * $P < 0.05$.

Figure S3. SiRNA-mediated inhibition of NFATc2 expression

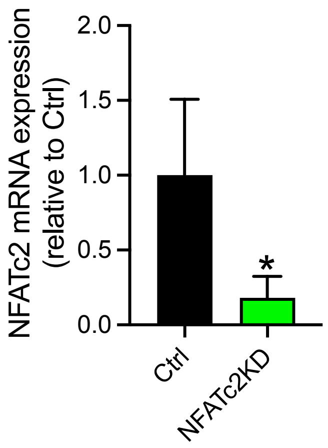

C2C12 myoblasts were transfected with control (Ctrl) or NFATc2 siRNA (NFATc2KD) and were maintained in GM for 24 h. Cells were then collected and processed for qRT-PCR or Western blot analysis. Fold changes of NFATc2 mRNA are shown. N = 6 per group. * $P < 0.05$. Data are presented as mean ± SD.

Table S1. Primer sequences used in qRT-PCR

	Primer sequence	
Target	Forward (5’-3’)	Reverse (5’-3’)
GAPDH	TGTGTCCGTCGTGGATCTGA	TTGCTGTTGAAGTCGCAGGAG
IL-4Rα	TCTGCATCCCGTTGTTTTGC	GCACCTGTGCATCCTGAATG
MyoD	GGACCCAGGAACTGGGATATG	TCATAGAAGTCGTCTGCTGTCTCAA
Myogenin	CAGTGAATGCAACTCCCACAG	TGGACGTAAGGGAGTGCAGA
Myomaker	ATCGCTACCAAGAGGCGTT	CACAGCACAGACAAACCAGG
Myomerger	CAGGAGGGCAAGAAGTTCAG	ATGTCTTGGGAGCTCAGTCG
MyHC4	GAGTTCATTGACTTCGGGATGG	TGCTGCTCATACAGCTTGTTCTTG
NFATc2	CACGGGCCAGAACTTCACAG	GCAAAGGCCGAGTCTCACAG
